# Compassionate Principlism: Towards a Novel Alternative to Standard Principlism in Bioethics

**DOI:** 10.1007/s11673-024-10373-9

**Published:** 2024-09-24

**Authors:** Adam J. Braus

**Affiliations:** Dominican University of California, 50 Acacia Ave, San Rafael, CA 94901 United States

**Keywords:** Principlism, Beneficence, Bioethics, Specification, Balancing, Reflective equilibrium

## Abstract

Principlism appears to be the prevailing applied ethical framework in bioethics. Despite the view’s various strengths, critics point out that since the principles are *ad hoc*, conflicts indubitably emerge leading to inconsistency. There is debate around whether principlism can provide definitive action-guiding moral prescriptions or only help structure intelligent analyses and justifications of moral choices. In this paper, I contend that applying concepts of *moral symmetry* and *moral asymmetry* allows us to modify one of principlism’s principles—the principle of beneficence—into what I will call the *principle of compassion*. I argue that the principle of compassion can function as an arbitrating or primary principle within the principlist framework. The result is a view we might call *compassionate principlism*. Arguably, compassionate principlism leads to fewer inconsistencies and provides more acceptable action-guiding moral prescriptions than traditional principlism.

## Introduction

Principlism (or the four principles approach to bioethics) is a pluralistic bioethical framework containing four key principles: justice, respect for autonomy, beneficence, and non-maleficence. Introduced by Thomas Beauchamp and James Childress (1979), the view appears to be the presiding paradigm in bioethics (Gillon 2015). That said, critics have noted methodological and practical problems, specifically when it comes to arbitrating between principles. Some argue that, because the principles are derived from contradictory ethical theories, they themselves are contradictory. The principles’ relationships to each other are *ad hoc* and unsystematic and indubitably will lead to conflicts (Clouser and Gert 1990; Davis 1995; Toulmin 1981; Walker 2009). Moreover, although principlism can frame intelligent debates about and justifications for ethical issues, it cannot provide definitive action-guiding prescriptions (Lee 2010; Hine 2011; Rigby and Symons 2023). To be action-guiding, principlism requires that one add supplementary values (opinions, beliefs, or the like) that are external to the four principles.

As we will see, principlism’s critics make some valid points. I therefore put forward a novel solution, one that can arguably deal with some of principlism’s inconsistencies. I will modify the principle of beneficence and contend that we can then use it to arbitrate conflicts between principles. This modification involves switching the principle of beneficence from being *morally symmetrical* to being *morally asymmetrical*. Because of its relationship to utilitarianism, the principle of beneficence is morally symmetrical. Moral symmetry sanctions trading the suffering of some for the happiness of others so long as it achieves the greatest net happiness. Moral asymmetry does not sanction trading the suffering of some for the happiness of others; suffering has lexically more “ethical weight.” I will call this asymmetrical version of the principle of beneficence the *principle of compassion*. For now, the principle of compassion can be roughly defined as follows:

*Act in a way that reduces unnecessary suffering as much as possible.*

I will hash out what the key term “unnecessary” means in Section “The Principle of Beneficence is Morally Symmetrical”.

I will argue that the principle of compassion can successfully arbitrate conflicts between principles. In doing so, we can maintain principlism’s practicality and flexibility while increasing its consistency and simplifying its application. This can, in turn, provide better analyses and justifications for the ethical practices of medical providers and researchers.

I will not try to provide a complete theoretical and/or ethical justification for the principle of compassion. It is also not possible to exhaustively demonstrate how the principle of compassion serves as an arbitrating principle in all cases. I will not attempt to prove that the principle of compassion always provides definitive action-guiding moral prescriptions. I will, instead, only argue that the principle of compassion appears to provide fewer inconsistencies and more definitive moral guidance than traditional principlism does in certain notable cases.^1^

Some might be tempted to think that I am simply trying to replace principlism with utilitarianism. This is not the case. Compassionate principlism should not confused with utilitarianism because it is morally asymmetrical. It also follows the principlist’s decisional procedure of methodically working through each principle. Note also that my goal is not to refute, denigrate, or replace principlism. Instead, I aim to strengthen the view by resolving the methodological inconsistency problem that critics have pointed out.

In Section "Principlism: Origins, Criticisms, and Responses", I briefly review and provide some background to principlism and how it has developed in the face of criticism. I will also review the literature on moral symmetry and asymmetry. In Section "Moral Asymmetry, Compassion, and Arbitration", I introduce and defend the principle of compassion. I argue that it functions well as an arbitrating principle because, with it, we can (1) explain (or account for) the other three principles, (2) set criteria for the limitations and justifiable infringements of the other three principles, and (3) satisfactorily arbitrate conflicts between the other three principles. In Section "Further Possible Objections", I will discuss some foreseeable limitations and challenges to my thesis.

## Principlism: Origins, Criticisms, and Responses

Each of principlism’s principles roughly corresponds to a prominent ethical theory or notion. Beneficence represents Bentham’s and Mill’s utilitarianism, non-maleficence represents Mill’s and Bernard Gert’s harm principle, justice represents John Rawls’ theory of justice, and autonomy represents Kant’s deontological view (Clouser and Gert 1990). Early critics claimed that principlism’s principles are inconsistent. As mentioned, the claim is that the principles are contradictory and *ad hoc* (nonsystematic), and there is no way to arbitrate between conflicting principles. Critics concluded that principlism cannot, on its own, provide definitive action-guiding moral prescriptions. K. Danner Clouser and Gert make the point as follows:

Since there is no moral theory that ties the “principles” together, there is no unified guide to action which generates clear, coherent, comprehensive, and specific rules for action nor any justification for those rules. (1990, 227)

The consequence is that principlist moral decisions must include outside values (opinions, beliefs, or the like) to arrive at definitive plans of action. Critics also claim that, on its own, principlism cannot discern whether someone is doing the right thing (Hine 2011). The view can only provide an intelligent framework for analyzing the relevant situation and justifying some course of action.

Some thinkers claim that *ad hocness* is actually one of traditional principlism’s strengths since it makes the principlist framework flexible enough to accommodate the demands of ethical pluralism and multiculturalism (Gordon 2011). Pluralism, they maintain, requires an applied ethics to be contradictory because some moral issues are unresolvable. Supporters of traditional principlism do, nonetheless, often claim to somehow arrive at action-guiding prescriptions (or at least recommendations). They must somehow arbitrate conflicts between principles (or risk Buridan’s ass-type dilemmas). Beauchamp and Childress suggest resolving such conflicts through a process of *specification*, *balancing*, and *coherent reflective equilibrium* (Beachamp 2007; Beauchamp and Childress 1979/2019). This process is not perfect, but it allows one to arbitrate conflicts and come as close as possible to definitive action-guiding ethical prescriptions. There is, though, still debate as to whether principlism provides action-guiding content or is more of a checklist that only reminds people to more fully apply their personal and cultural moral intuitions (and biases) (Hine 2011).

Initially, some considered the principle of autonomy to be an arbitrating or primary principle among the four. A notable response is, however, that this represents a kind of Western cultural imperialism (Gillon 2015). Respect for individual autonomy cannot serve as an arbitrating principle because it represents an Anglo-American cultural bias. Some cultures place more emphasis on an individual’s connectedness to family, community, and the nation than on autonomy (Huxtable 2013, 40). Principlists have largely abandoned elevating autonomy to the status of arbitrating principle, and now tend to think of the principles as coequals (Gillon 2015; Hammersley 2015, 445; Beauchamp 2007, 8). Raanan Gillon considers this to be a good thing. He writes:

The fact that principlism in itself does not provide a universalizable method of prioritizing the four principles, far from being a fatal blow to principlism is on the contrary a major advantage. (Gillon 2015, 113)

Given ethical pluralist and multiculturalist concerns, some have, then, come to think of inconsistency as a strength (even if it was initially thought of as a methodological weakness).

Beauchamp and Childress theoretically grounded principlism in Gert and Clouser’s theory of common morality, according to which there is an intuitive, universal, and rational basis for right and wrong. B. Andrew Lustig (1992) has proposed a way to weigh or balance which principles deserve more or less consideration in any given situation via a kind of moral intuition. David DeGrazia (1992) claims that the four principles need not be systematically related at all. We are supposed to work through the four principles like a checklist. As someone works down the list, they will expose and unearth considerations that together constitute a kind of reflective equilibrium. One can then judge whether one’s derived equilibrium is correct by assessing its coherence.

Beauchamp and Childress call their solution to the problem of inconsistency “specified principlism.” Here, the principles are only *prima facia* principles. The idea is that, through specification and balancing, one can reach a reflective equilibrium that (all things considered) leads to a justifiable ethical judgment. This procedure putatively allows us to work out what our moral obligations are. We cannot be certain, but the principles afford us the “highest level of certitude” we can have (Davis 1995, 439).

Beauchamp defines “specification” as “a process of reducing the indeterminateness of abstract recommendations and providing them with specific action-guiding content” (2007, 8). When working with mentally incompetent patients, for example, we must specify the principle of respecting patient autonomy: “Respect the autonomy of competent patients by following their advanced directives when they become incompetent” (Beauchamp and Childress 1979/2019, 17). Specification reduces indeterminacy regarding how to apply a principle to a specific scenario, but it does not solve the problem of contradictions between principles. Two specified principles can contradict each other if they are inherently inconsistent.

Beauchamp and Childress thus introduce balancing, which they define as “the process of reasoning about which moral norms should prevail when two or more of them come into conflict” (1979/2019, 20). They describe a physician having to decide whether she should stay late at work to care for a patient in need or take her son to the library as promised. According to Beauchamp and Childress,

… [s]he engages in a process of deliberation that leads her to consider how urgently her son needs to get to the library, whether they could go to the library later, whether another physician could handle the emergency case, and the like. (1979/2019, 20)

If the patient is likely to die without the physician’s help, then the patient’s life weighs more on balance than going to the library.

Balancing enables “reaching judgements in *particular cases*” and specification enables “developing more *specific policies* from already accepted general norms” (Beauchamp and Childress 1979/2019, 20). The process of specification and balancing allows one to develop a (coherent) reflective equilibrium between principles. Beauchamp and Childress are sceptical that we can ever gain absolute ethical knowledge. They state that “[i]n our judgement, no general moral theory has ever achieved the goal of a fully specified system of norms for health care ethics” (Beauchamp and Childress 1979/2019, 430). They are satisfied with a framework that provides some kind of structure, even if it contains a “measure of disunity, conflict, and ambiguity” (Beauchamp and Childress 1979/2019, 431). These, they say, are simply “pervasive features of the moral life” (Beauchamp and Childress 1979/2019, 431).

Nonetheless, a question remains as to whether or not principlism contains action-guiding content (see notably Hine 2011). It appears contradictory when Beauchamp, for instance, often states that the principles are flexible and *prima facie* incomplete, but, at other times, states that principlism requires a physician to follow some specific action (e.g. overriding the wishes of a Jehovah’s Witness parent by providing a life-saving blood transfusion to her child) (Beauchamp 2003, 271).

Brieann Rigby and Xavier Symons (2023) have discussed cases where various supporters of principlism stipulate moral prescriptions regarding abortion and voluntary euthanasia. If principlism is only a way to structure analysis, then it cannot demand any moral action in and of itself. However, if the principlist framework does provide definitive moral guidance, then it is vulnerable to the methodological criticisms discussed above. Some have, consequently, suggested that principlism should be restricted to constituting a framework for organizing analyses and justifications, and should not be used as a method for uncovering binding moral obligations (Lee 2010; Hine 2011; Hammersley 2015; Rigby and Symons 2023).

## Moral Asymmetry, Compassion, and Arbitration

So far, we have established that principlism faces, at least, one salient methodological problem: Inconsistency between the principles, leading to conflicts. It is not at all clear that specification, balancing, and forming a coherent reflective equilibrium satisfactorily resolve such conflicts without invoking the problems discussed in the previous section. One solution would be to find an arbitrating or primary principle, one that can resolve conflicts between the other principles. As mentioned, respect for autonomy was put forward as an arbitrating principle, but doing so invokes worries related to cultural imperialism.

In this section, I will argue that we can establish a new arbitrating principle by modifying the principle of beneficence. This modification involves transforming it from being morally symmetrical to morally asymmetrical. For reasons that will become clear, I am calling the principle of asymmetrical benevolence the principle of compassion. To be clear, “the principle of compassion” is synonymous with “the principle of asymmetrical beneficence.” I am suggesting that the principle of beneficence be transformed into the principle of compassion. We are then left with four principles: (1) compassion, (2) justice, (3) non-maleficence, and (4) autonomy. Compassion serves as the arbitrating principle.

In making my case, I will, firstly, justify modifying the principle of beneficence from being symmetrical to asymmetrical. Secondly, I will explain (or account for) the other three principles in terms of the principle of compassion and also define their limitations. Thirdly, I will argue that the principle of compassion allows us to arbitrate conflicts arising between the other three principles. As we will see, Beauchamp and other supporters of traditional principlism are, in many cases, already intuitively using something like the principle of compassion to solve conflicts between principles. They do so by picking the most compassionate course of action, namely the one that leads to the least unnecessary suffering.

### Moral Symmetry Versus Asymmetry

As mentioned in the introduction, moral asymmetry does not sanction trading the suffering of some for the happiness of others. Suffering and happiness are separate and non-convertible. Karl Popper put it this way:

There is, from an ethical point of view, no symmetry between suffering and happiness, or between pain and pleasure. [...] In my opinion, [...] human suffering makes a direct moral appeal, namely, the appeal for help, while there is no similar call to increase the happiness of a man who is doing well anyways (1945/2020, 602; Tranöy 1967, Mayerfeld 1996, and Wolf 2004 express a similar view).

Jamie Mayerfeld (1996) calls this kind of relationship between suffering and happiness *moral asymmetry*. Its moral intuition relies on the claim that reducing suffering is of greater moral concern than increasing pleasure or happiness.

David Parfit defends a similar idea via his “Claim about Compensation,” namely, that “from a moral point of view, pain cannot be outweighed by pleasure, and especially not one man’s pain by another man’s pleasure” (1982, 337; see also Wolf 2004, 63). Moral asymmetry suggests both (a) that we cannot compensate suffering with happiness across individuals and groups and (b) that reducing suffering is more morally important than increasing happiness. Or in other words, if we are given the choice, we are obligated to feed a starving man before feeding a fat one. The idea of moral asymmetry is controversial and stands in contrast to several prominent ethical theories. In standard utilitarianism, the suffering of some is sometimes necessary to reach the greatest good. For deontologists, inflicting suffering might be required when performing one’s duty. Nevertheless, the intuition that our moral obligations to help those who are suffering trump obligations to help those who are happy is intuitively compelling (I will return to this topic in Section “The Principle of Compassion (aka Asymmetrical Beneficence”). This view is relatively underdeveloped and merits further research.

### The Principle of Beneficence is Morally Symmetrical

The principle of beneficence is morally symmetrical because it is derived from utilitarianism, which is morally symmetrical. Utilitarian ethical theories explicitly sanction making tradeoffs between the suffering of some and the happiness of others (as long as doing so leads to a net increase in happiness). Following Bentham, utilitarians might claim that “it is the greatest good to the greatest number of people which is the measure of right and wrong” (1776/1970, 393). Utilitarians (at least the vanilla variety) treat increasing happiness and reducing suffering as reciprocal, compensatory, and therefore symmetrical. As Mill puts it, “actions are right in proportion as they tend to promote happiness, wrong as they tend to produce the reverse of happiness” (1863/2001, 7). If so, then increases in happiness can compensate for increases in suffering. Indeed, making such compensatory tradeoffs is sanctioned and even required. Beauchamp follows this utilitarian motif by defining the principle of beneficence as morally symmetrical because it requires both increasing positive welfare and reducing negative welfare: “This principle includes rules such as ‘maximize possible benefits and minimize possible harms’ and ‘balance benefits against risks’” (Beauchamp 2007, 5).

There are various reasons why we can justifiably question basing the principle of beneficence on moral symmetry and instead entertain the idea of changing it to moral asymmetry. Firstly, moral symmetry leads to objectionable moral outcomes in our current medical and research system. For example, under moral symmetry, the rise of a pharmaceutical company’s stock price (which enriches its executives and investors) must (at least partly) counterbalance the suffering caused by that company’s research priorities, which favour treating diseases of the affluent and neglecting diseases of the poor. Secondly, under moral symmetry, the rise in a for-profit health insurance company’s stock price (at least partly) outweighs the suffering of working, rural, and poor people who cannot afford health insurance. These trade-offs are necessary outcomes of a symmetrical version of beneficence. But, they are morally objectionable if (as I am contending here) the increased happiness of some does not compensate for the avoidable misery of others.

Beauchamp and likeminded principlists define the principle of beneficence morally symmetrically. Yet, when listing examples of this principle, they only discuss morally asymmetrical instantiations and overlook morally symmetrical ones (at least in the literature I have encountered). As an example of the principle of beneficence, Beauchamp, for instance, includes the “assistance of those in need of treatment or in danger of injury” (2007, 5). Moreover, the “harms to be prevented, removed, or minimised are the pain, suffering, and disability of injury and disease” (Beauchamp 2007, 5). Likewise, when providing examples of beneficence’s “range of benefits,” Beauchamp lists cases of reducing suffering. These include “helping patients find appropriate forms of financial assistance and helping them gain access to health care or research protocols” (Beauchamp 2007, 5). Beauchamp does not include symmetrical examples of beneficence, such as advances in performance-enhancing drugs, enhancements related to longevity, advances in cosmetic surgery, increases in the revenue of medical companies, disproportional research dollars going to fight the diseases of the affluent, the prestige for doctors and institutions, or the like.^2^ These are salient features of the present medical system in the United States and globally. If beneficence were morally asymmetrical, then then these pursuits would have no moral weight at all, but they clearly weigh on the decision making of hospitals, research organizations, and governments.

The ambiguity between a morally symmetrical definition and morally asymmetrical examples of the principle of beneficence is a significant problem for standard principlism. Should the principle of beneficence be morally symmetrical or asymmetrical? It cannot be both. Here we can make this remarkable ambiguity clearer and explore what an explicitly moral asymmetrical version of the principle of beneficence would look like and how it would operate.

There is further justification in proposing a morally asymmetrical version of the principle of beneficence in observing that the moral obligations of medical providers and researchers are intuitively morally asymmetrical. Medical researchers and providers have a significant moral obligation to treat the sick and comfort the dying. They do not have a corresponding (or symmetrical) moral obligation to make the healthy more well. Intuitively, elective sorts of medicine and research (e.g. cosmetic dermatology and performance enhancement) have less moral weight than life-saving medicine. Indeed, if a cosmetic surgeon tries to morally defend her kind of medicine, then she will most likely point out that, for many of her patients, the procedures help reduce body dysmorphia, depression, or similar types of suffering. Rather than being a refutation, this line of argument strengthens the case for moral asymmetry in bioethics. The cosmetic surgeon’s work becomes more morally defensible the more she can demonstrate that it is morally asymmetrical.

### The Principle of Compassion (aka Asymmetrical Beneficence)

Given the above, we are both justified in doubting a morally symmetrical principle of beneficence and in exploring the possibility of exchanging it for an asymmetrical version. In this regard, the principle of asymmetrical beneficence—or the principle of compassion^3^—is justified in being considered sufficiently new and as deserving special definition and attention. Such a principle might stipulate as follows:

*It is morally obligatory to reduce unnecessary suffering but only morally praiseworthy to increase happiness.*

Put otherwise, the principle of compassion encourages reducing *unnecessary suffering* as much as possible. Neither Popper nor other supporters of moral asymmetry have clearly defined what the term “unnecessary suffering” (or its cognate “avoidable misery”) means. For the purposes of this paper, I take it that most readers will have an intuitively satisfactory understanding of what “suffering” or “misery” means. We can, though, define “*unnecessary* suffering” as follows:

An involuntary suffering that we are capable of reducing and our attempt to reduce will not likely cause more misery than enduring it.

Thus, an avoidable misery (or unnecessary suffering) is a misery that (a) is a real and factual misery rather than fraudulent, (b) someone has not chosen to experience it (it is involuntary or nonconsensual), (c) we have the capacity to reduce it, and (d) the steps to reducing it will not cause some greater misery (it is not the “least worst option”). Conversely, an unavoidable misery (or necessary suffering) is a misery that is either (a) fraudulent, (b) voluntary, (c) we are not capable of reducing it, or (d) attempting to reduce it is likely to lead to a net increase in misery (it is the “least worst option”). Each of these criteria outlines the kinds of misery that call for urgent attention and remedy.

- Being a *real* misery matters because a fraudster can mislead people by claiming that some terrible suffering is occurring when it is not.
- *Consent* matters because consensual acts (consensual sex, exercise, dieting, labour, and the like) are morally acceptable. But, the lack of consent renders them immoral actions (rape, coercion, starvation, slavery, and the like).
- *Capacity* matters because (all things considered) we cannot be obligated to do what we are not capable of doing, or *ad impossibilia nemo tenetur* (ought implies can). Hence, miseries that fall outside of our ability to change can be labelled as necessary or unavoidable.
- Not being the *least worst option* matters because, if the goal is to reduce misery, then it would be (all things considered) absurd for us to be obligated to take actions that eliminate misery when those actions ultimately cause a net increase in misery.

For an example of the difference between avoidable and unavoidable misery, we can compare teen heartbreak to teen pregnancy. Teen pregnancy is a form of unnecessary suffering because sexual education and access to contraception do not cause more misery than teen pregnancy. Teen heartbreak, in contrast, is an unavoidable misery because the interventions preventing teenagers from falling in love with each other will be more miserable than simply allowing it to occur. Imagine segregating romantically attracted people (heterosexuals from the opposite sex and homosexuals from the same sex), propaganda campaigns about the dangers of fraternization and romance, punishments for those who do fall in love, and the like. *Ex hypothesi*, we have a more urgent moral obligation to reduce teen pregnancy and only the praiseworthy option to reduce teen heartbreak.

Not everyone will agree with my proposed definition of “avoidable” misery. There are, no doubt, edge cases that challenge its completeness. Although a fuller discussion of this definition is necessary, it is not my goal here to strictly defend one definition. My goal is only to provide a plausible working definition so that we can continue with a discussion of the principle of compassion (and ultimately outline my proposed novel view: compassionate principlism).

A possible objection to applying moral asymmetry to the principle of beneficence is that this has already been done in Gert’s critique of principlism and his application of the theory of common morality. But, this is not correct. Although the theory of common morality favours reducing harm over increasing benefits, it is still morally symmetrical. Gert and colleagues define “harm” as “unmitigated harm” (i.e. harm that is not mitigated by some benefit) (see, e.g., Gert et al. 2006, 8). Common morality (as Gert and Clouser define it) allows benefits to outweigh harms. This renders the view as morally symmetrical as standard utilitarianism or principlism’s principle of beneficence. Whether the theory of common morality could or should be updated to be morally asymmetrical is, though, outside of the scope of this discussion.

### How the Principle of Compassion can Arbitrate Conflicts

We can use the principle of compassion to arbitrate various conflicts between the other principles. I contend that this represents a process that is (at least in some cases) more definitive, coherent, and therefore satisfactory than the standard principlist process involving balancing and reflective equilibrium.

We begin the same way, by walking through the four principles and making any requisite specifications. If any ambiguities or conflicts arise between the principles, then we can take recourse to the principle of compassion. We ask “Which course of action will lead to the most compassionate outcome?” In other words, what is the course of action that will lead to the least unnecessary suffering (or avoidable misery)?

As mentioned, critics have argued that principlism (in its current form) is too inconsistent to provide action-guiding prescriptions or recommendations. It is not clear whether compassionate principlism would meet all definitions of “consistency” found in the literature (e.g. Lee 2010; Hine 2011; Huxtable 2013; Hammersley 2015; Rigby and Symons 2023). Nevertheless, compassionate principlism is more coherent and less arbitrary than standard principlism. We can see this if we return to the example of a child of Jehovah’s Witnesses who needs a blood transfusion. Beauchamp claims that principlism requires overriding the parent’s wishes. Compassionate principlism comes to the same conclusion but in a more coherent and philosophically systematic way. Remember that an avoidable misery must be involuntary. So, if an adult Jehovah’s Witness refuses a blood transfusion for herself, then the consequent misery would be voluntary and thus unavoidable. The doctor cannot ethically force a blood transfusion onto the patient. There can always be extenuating circumstances (such as a patient being impaired or in shock), but I am only concerned with generic cases here. In any event, a parent is not their child; they are only the child’s guardian (assuming that it is a young child). The child is not choosing to deny the blood transfusion. Thus, for the child, the blood transfusion is ethically required. The doctor must override the parent’s religious preference.

There can, of course, be other considerations. An official policy overriding religious preferences might make the entire Jehovah’s Witness community (and a few others) abandon modern medicine entirely, thereby causing even greater misery. Such questions must be considered. But, notice that this is an epistemic question about the best way to reduce avoidable misery rather than an ethical question about which moral principle or theory is on balance more weighty or more important. If we can move from a moral debate to an epistemic one then we have, at least, succeeded in finding moral agreement.

It is important to note that, while making theoretical arguments to defend standard principlism, Beauchamp and Childress already appear to be, unself-consciously, employing the principle of compassion to arbitrate conflicts between the principles. But, in the “real world,” standard principlism sanctions less than compassionate outcomes. In their discussion of specification, for example, Beauchamp and Childress point out that “some specified recommendations are virtually absolute and need no further specification” (1979/2019, 19). In other words, Beauchamp and Childress claim that the rightness or wrongness of some actions are so obvious they do not require any specification, and presumably they do not require balancing either. The examples they cite are almost verbatim expressions of the principle of compassion. They state, for instance: “Examples include prohibitions of cruelty that involve unnecessary infliction of pain and suffering” (Beauchamp and Childress 1979/2019, 19). Elsewhere, Beauchamp states that we can

… justifiably not tell the truth in order to prevent someone from killing another person, and we might justifiably disclose confidential information about a person in order to protect the rights of another person. (2007, 7)

This claim—presumably arrived at via balancing and reflective equilibrium—is, in short, a more roundabout way to simply applying the principle of compassion. In each case, one chooses the plan of action that will lead to the least unnecessary suffering. However, when we examine the real-world outcome of applying standard principlism to medical research and medical care, we tend to find notable inequities and unnecessary suffering.

Medical care or research institutions (whether for-profit, non-profit, or governmental) generally have ethics boards with significant sway over the actions of that institution. These boards primarily use the standard principlist method for justifying moral choices. Indeed, this method arguably dominates the field of bioethics (Lee 2010; Rigby and Symons 2023). We can, then, think of the current circumstances in medical care and research as being (at least to some significant degree) justified by principlist motifs. Yet, there are significant ethical problems in healthcare and medical research. In the United States, private health insurance, the fee-for-service medical care system, and for-profit pharmaceutical companies leave a large percentage of working, rural, and poor people without access to or bankrupted by medical care. In global medical research, the world suffers under the “10/90 gap,” which refers to the fact that only 10 per cent of health research dollars go toward solving health problems suffered by 90 per cent of the global population (Campbell 2017, 137). In medical care, global development data suggests that the world also suffers under the “inverse care law,” which states that the “availability of good medical care tends to vary inversely with the need of the population served” (Watt 2002). In other words, research funding and medical care are largely directed toward diseases that affect the wealthy and powerful rather than diseases that affect everyone else. Since standard principlism is the ethical theory that predominates bioethics itself, the glaring, persistent, and inequities in the conduct of medicine reflects poorly on the framework. It is not the goal here to judge the actions or moral attitudes of the members of medical ethics boards (it is likely they are also not pleased with these inequities!). Rather the goal here is to suggest that standard principlism theoretically sanctions morally objectionable outcomes like our present problems with medical inequity.

In the case of persistent medical and research inequity, the principle of justice conflicts with the principle of symmetrical beneficence in standard principlism. In this conflict, the principle of symmetrical beneficence appears to be “winning” during the process of specification and balancing because medical institutions are tolerating the avoidable injustice. During balancing, the increased health and welfare of the already well-off is outweighing the justice of providing equitable healthcare to those who are not well-off. Again, I am not saying that this is anyone’s opinion or belief *per se*, only that the framework sanctions this argument in theory. Standard principlism allows for balancing to be relatively arbitrary. Increased symmetrical beneficence (or any other principle) can outweigh another principle (in this case justice) and result in additional needless suffering. As argued, we can avoid this scenario if we apply the principle of compassion as the arbitrating principle when deliberating the kinds of conflicts I have been discussing.

Under compassionate principlism, increasing the health or wealth of the healthiest and wealthiest does not weigh on the scales of morality; only increasing the health of the ill does (irrespective of their wealth or power). In this sense, a compassionate form of principlism is in better alignment with the attitudes of people who would see inequities in medical care and research as morally intolerable than standard principlism. It would be extremely challenging to justify the pursuance of a national or global health regime as inequitable as our current one. Would that mean, in practice, that things like the 10/90 gap in medical research funding and the inverse care law would vanish? It is impossible to say since the practical circumstances are quite complex, but theoretically, at least, these outcomes would be morally untenable. Ethically speaking, it would be a foregone conclusion that powerful institutions and government must (a) work to provide universal healthcare and (b) dedicate funds and efforts to medical research that promises the greatest reduction in avoidable misery regardless of financial situation or geopolitical power.

### The Principle of Compassion is Different from the Others

One objection to elevating the principle of compassion into the primary and arbitrating principle is that the decision is arbitrary, and we might as well put any of the four principles in this position. To this objection we must contrast the principle of compassion with the other principles. We shall see that the principle of compassion has at least two unique features that single it out as suitable to arbitrate among the others. A) Infringing on any of the other three principles infringes either directly or instrumentally on the principle of compassion and B) one can use the principle of compassion to define satisfactory criteria for when it is justifiable to infringe on the other three principles; namely, infringements of other principles are allowed if and only if that action leads to the least unnecessary suffering. None of the other principles have these traits. To make my case, I will discuss the principles of non-malfeasance, autonomy, and justice in turn.

#### The Principle of Non-malfeasance

When one infringes on the principle of non-malfeasance one also infringes on the principle of compassion since one increases the total amount of unnecessary suffering. The principle of non-malfeasance is synonymous with the Hippocratic Oath (viz. “*premium non nocere*” or “above all, do no harm”) (Beauchamp 2007). It requires doctors to avoid acts that harm their patients or work against their patients’ interests. Examples include selling or prescribing medicine with bogus or unproven effects, changing a patient’s treatment for personal gain, or (more obviously) knowingly harming a patient. Each of these cases would cause harms which would constitute an increase in unnecessary suffering and therefore infringes on the principle of compassion.

There are occasions when it is morally acceptable to harm medical patients or research subjects, and we can (and already do unconsciously) use the principle of compassion to define theses occasions. For instance, when proper anaesthesia is absent (say on a desert island), a doctor is still morally required to conduct a life-saving procedure, even if it will be very painful. Another case where harm is permitted is when conducting research where consenting subjects must endure some harm or discomfort. As these cases suggest, it is acceptable to initiate harm in cases in which doing so prevents even greater unnecessary suffering. For instance, enforced quarantines are harmful insofar as they are psychologically uncomfortable, but they are morally obligatory if they avoid greater suffering from the spread of a disease. Hence, we can (and already do) use the principle of compassion as the criteria for when it is permissible to infringe on the principle of non-maleficence. None of the other principles define the criteria for infringing on another principle. This unique trait singles the principle of compassion out as a suitable candidate for an arbitrating principle among the four.

#### The Principle of Autonomy

When we infringe on the principle of autonomy we also, either directly or instrumentally, infringe on the principle of compassion. Losing autonomy over oneself, one’s family, and one’s community causes individuals to lose their integrity and their dignity which causes great distress and suffering. For example, when paternalistic systems, policies, or experts deny people’s autonomy, they infringe on those people’s dignity (and the dignity of their families and communities). Lying to patients, withholding important information, and the like are all infringements on people’s integrity and dignity and are a direct or instrumental causes of profound suffering. Take an elderly person suffering from cancer. Anomalous cases aside, a doctor withholding the patient’s diagnosis out of a misguided attempt to reduce her distress counts as a condemnable action. All things being equal, withholding the information causes the greater harm of diminishing the person’s integrity and dignity. Failing to respect the autonomy of individuals and the self-determination of families and other groups leads to unnecessary suffering.

There are, however, cases where we might be required to override someone’s autonomy. To establish criteria for when we are permitted to deny someone’s autonomy, we can turn to the principle of compassion and ask, “Which course of action will lead to the least avoidable misery?” In practice, these calculations will often be complex, requiring extensive research and debate. In principle, however, appealing to the principle of compassion represents a potentially workable and theoretically sound method for discerning what to do in the relevant cases. For instance, in cases of attempted suicide, we are often required to infringe on the suicide’s autonomy to stop them from killing themselves (even by force). Likewise in the case of a hypochondriac or drug seeker, we are required to interfere with their desires and preferences for physical care (and we might instead recommend psychological care). This suggests that (as with non-malfeasance) the principle of compassion is a workable principle for defining the limits of other principles.

#### The Principle of Justice

Like autonomy, intolerable infringements of the principle of justice cause, either directly or instrumentally, an increase in unnecessary suffering. Imagine that a junta seizes a farm and kicks out the family. It is miserable to have one’s integrity unjustly trod upon or to watch it happen to others, but an initial injustice is likely to lead to countless other miseries. The victim’s dignity is reduced, they fall into poverty, their health suffers, their family suffers, and all the social ills of poverty befall them. Perhaps they seek retribution which sparks a cycle of violence. Morally intolerable forms of injustice can destabilize the order of society itself, which would lead to myriad miseries. There are many cases of medical injustice. There is, for instance, an unfair and inequitable distribution of access to healthcare and affordability of health insurance in the United States.^4^ Even those with health insurance and access to healthcare can be victims of injustice. An example is well-documented racial and ethnic disparities in U.S. organ donation practices (Park et al. 2022). These injustices compound the suffering and hardship of an already structurally disadvantaged group. Of course there are just forms of redistribution that are morally tolerable. These, I would suggest here, are forms of redistribution that minimize avoidable misery, for instance taxation of the have’s to provide basic services for the have not’s. The morally intolerable forms of injustice are also infringements on the principle of compassion.

There are, of course, occasions when we seem morally required to treat people unequally, and in that sense unfairly. By invoking the principle of compassion, we can define criteria for when injustice or unfairness seems to be required. Unfairness can be required if the outcome is to reduce unnecessary suffering. An example relates to the justice or injustice of taxation. If the money of certain taxes goes to waste or is used to increase suffering, then those taxes represent an increase in avoidable misery and is immoral. But, if taxes are spent to reduce unnecessary suffering (e.g. to make healthcare accessible to and affordable for the disadvantaged), then taxation can count as morally permissible. Another example would be lifeboat scenarios in which one must make tragic, unfair (perhaps even cruel and morally painful) decisions to prevent the worst-case scenario.

Some might object that, if any infringement of the other principles also infringes on the principle of compassion, then there is no reason to have the other principles at all. In response, I maintain that the other principles still have practical value. As both supporters of principlism and its critics agree, the principles today serve very well as organizing structures for ethical analyses and justification (Section "Principlism: Origins, Criticisms, and Responses"). Principlism’s popularity suggests that scholars and practitioners find the principles useful and easy to deploy in this practical sense. The other principles also highlight subtle (even invisible) forms of unnecessary suffering, forms that might otherwise be forgotten or ignored. For instance, respecting others’ autonomy might appear obvious to many, yet the notion of individual autonomy can be quite subtle. There are cultures with paternalistic medical practices or authoritarian decision-making systems. These cultures risk neglecting individual autonomy and therefore people’s integrity and dignity. Likewise, in highly individualistic cultures, medical practitioners might have difficulty recognizing the autonomy of groups such as families and communities and run the risk of damaging the dignity and integrity of members of those groups. Similarly, the principle of justice can be so subtle that, even when explicitly stated, people might fail to consider the impact their actions have on justice, so it is valuable to call out and highlight each principle specifically even if it is technically included in compassion. As I discussed above, medical care and research are distributed very inequitably (not only in the United States but also globally) despite the principle of justice’s current inclusion in the principlist framework.

## Further Possible Objections

There are various foreseeable questions about or rebuttals to my suggestion that compassionate principlism replace standard principlism. In this section, I anticipate three possible objections and then respond to each.

### Objection 1

Some might object that no principle can be elevated above the others within the principlist framework without leading to some kind of cultural imperialism. As mentioned before, some principlists attempted to elevate the principle of autonomy to the status of an arbitrating principle. Some writers criticized this move claiming that that it was a form of cultural imperialism. Treating principle of compassion as an arbitrating principle will necessarily mean another kind of imperialism. As we saw, compassionate principlism forbids a Jehovah’s Witness from denying her child a blood transfusion. Compassionate principlism sanctions paternalistic or imperialistic types of medicine and research.

Compassionate principlism does indeed requires altering cultural practices that lead to unnecessary suffering. But, this does not constitute the imperialistic elevation of one culture over another. Compassion is found across cultures. It is not owned by any one culture. The principle of compassion accommodates multiculturalism because denying or erasing someone’s culture can cause much suffering. The suffering of infringing or contradicting someone’s cultural beliefs and practices must be included in our analyses of right and wrong. In this sense, the principle of compassion obligates us to protect cultural practices (related to, e.g., language, dress, religious beliefs, and livelihood) if they do not lead to unnecessary suffering. Compassionate principlism opposes cultural practices that leads to unnecessary suffering. Plausible examples include child marriage, female circumcision, and the persecution of supposed witches. Surely, only the most fanatical multiculturalists will believe that these practices are ethically tolerable. One of compassionate principlism’s strengths is, then, that invoking the principle of compassion allows us to (in principle) delineate between which cultural beliefs we can police and which we must uphold (or at least tolerate).

### Objection 2

Another possible objection to establishing an arbitrating principle is that doing so appears to support a kind of moral universality when Beauchamp and Childress have explicitly stated that no ethical theory can be universal (1979/2019, 430). One ethical theory could not explain the variety and depth of moral attitudes found around the world. There are so many different moral attitudes, often held with a steadfast conviction. It is highly unlikely that one principle or ethical theory could encompass all such attitudes in all scenarios.

In response to this objection, I recognize that it might be impossible to say whether or not we will ever find a truly universal ethical theory or framework. In practice, all we can do is conduct research, propose improvements, and engage in lively debates. The theories and principles emerging out of this process should, though, at least become better over time (where “better” here means we can identify some general, albeit piecemeal, moral progress). If my overarching argument is on point, then compassionate principlism offers methodological and practical improvements over standard principlism. There is, however, no way to know if the theory is truly universal since there will always be unforeseen ethical dilemmas and new developments as medical, biological, and bioethical research continue to develop.

## Conclusion

I have presented a novel proposal aimed at addressing certain methodological and practical challenges inherent in the principlist framework. Despite its flexibility, popularity, and practicality, principlism seems to suffer from problems of methodological inconsistency. Despite principlists’ efforts to develop a procedure for resolving conflicts, it is doubtful whether their framework can offer definitive action-guiding moral prescriptions.

In attempting to bolster principlism’s coherence and efficacy, I argued that we can elevate a modified version of the principle of beneficence to a primary arbitrating position. This modification entails a conceptual shift from symmetrical to asymmetrical beneficence. Asymmetrical beneficence—otherwise known as compassion—rejects the trading suffering for happiness across individuals or groups. Instead, it requires minimizing unnecessary suffering (or avoidable misery).

By making the principle of compassion an arbitrating principle, we can maintain principlism’s pragmatic and adaptable nature. But, compassionate principlism offers a more coherent and efficacious method for reconciling conflicts between the principles, namely, taking recourse to the principle of compassion. This, in turn, provides a more robust foundation for both ethical analyses and decision-making in medicine and research.

I have not offered an exhaustive ethical justification for the principle of compassion or a thoroughly worked out explication of compassionate principlism (*qua* ethical theory). My primary aim has been to demonstrate the principle of compassion’s potential as a guiding principle within the principlist framework. Further explorations and refinements of this notion are surely required, including further examination of its limitations. Nevertheless, I hope to have shown that compassionate principlism represents a promising option for fortifying the presiding bioethical framework. By embracing the principle of compassion as a central guiding tenet, we can potentially navigate intricate ethical quandaries with heightened clarity and sensitivity. This can, in turn, advance a more ethically informed and (of course) compassionate approach to healthcare and medical research.

## Data Availability

Not applicable.

## Footnotes

1 What I call here standard or traditional principlism is best summed up and represented by Beauchamp and Childress (1979/2019)

2 For more examples of supporters of principlism using asymmetrical examples of beneficence, see also Lindridge 2017, 10; Bulger 2007, 93; DeMarco 2005, 104.

3 One might also refer to this principle as “*misericordia*” after the Latin word for “compassion” or “pity.”

4 Even those who are against a more equitable system of healthcare do not tend to deny that the current system is unfair and inequitable. They might, instead, attempt to justify the inequity as being unavoidable because, in their assessment, it services some greater good (or the present system is the least worst option).
